# The Influence of Irisin on Selected Organs—The Liver, Kidneys, and Lungs: The Role of Physical Exercise

**DOI:** 10.3390/cells14161228

**Published:** 2025-08-08

**Authors:** Maria Ciałowicz, Marek Woźniewski, Eugenia Murawska-Ciałowicz, Piotr Dzięgiel

**Affiliations:** 1 Department of Physiotherapy in Internal Diseases and Oncology, Wroclaw University of Health and Sport Sciences, 51-612 Wrocław, Poland; 2 Department of Physiology and Biomechanics, Wroclaw University of Health and Sport Sciences, 51-612 Wrocław, Poland; 3 Department of Human Biology and Cosmetology, Wroclaw University of Health and Sport Sciences, 51-612 Wrocław, Poland; 4 Department of Human Morphology and Embryology, Wroclaw Medical University, 50-368 Wrocław, Poland

**Keywords:** irisin, physical exercise, liver, kidney, lungs

## Abstract

In recent years, irisin has garnered significant interest among researchers. It is a myokine released by skeletal muscles during physical exercise. Its expression occurs not only in skeletal muscles but also in other organs such as the liver, kidneys, and lungs, where it fulfills important metabolic and protective functions. Irisin is involved in the regulation of energy homeostasis, promotes the browning of adipose tissue, plays a protective role, and influences the body’s adaptation to physical exercise. In the context of internal organ function, studies suggest its potential role in protecting the kidneys from damage, modulating inflammatory processes in the lungs, and supporting liver regeneration. This literature review focuses on analyzing the therapeutic effects of irisin in these organs in relation to the role of physical exercise.

## 1. Introduction

From a physiological standpoint, all benefits arising from regular physical activity, such as the optimization of energy metabolism through a balance of anabolic and catabolic processes, stem from the crosstalk between organs [1]. Tissues and organs playing key roles in energy metabolism regulation include adipose tissue, liver, brain, and muscle tissue, forming a system of interactions regulated by the central nervous system [2].

The favorable impact of physical exercise on overall body function is evident in a wide range of adaptive changes. Among the numerous effects stands out the role of exercise in regulating metabolic processes at different organizational levels, including the tissue, cellular, biochemical, and molecular levels. Hormones are also involved in metabolic and energetic regulation. In addition to the well-known most important hormones that influence energy metabolism, such as insulin, glucagon, cortisol, growth hormone, and androgens, substances called organokines play a significant role. These possess auto-, para-, and endocrine functions [2]. Organokines are secreted by various tissues. Skeletal muscles secrete myokines, white adipose tissue secretes adipokines, brown adipose tissue produces batokines, the liver secretes hepatokines, and bone tissue releases osteokines. Organokines secreted by the nervous system are known as neurokines, and those secreted by the heart as cardiokines [2,3,4,5]. Among the organokines is irisin, which serves similar functions to myokines, neurokines, hepatokines, osteokines, and adipokines.

Skeletal muscles, contracting in response to physical activity, release a large group of peptides known as myokines. These myokines can influence not only muscle tissue function but also a wide array of other tissues and organs, including adipose tissue, liver, brain, bones, and kidneys [6]. Irisin is also referred to as the “exercise-induced protein.” Physical activity promotes the regulation and secretion of various organokines, forming a network that facilitates the crosstalk between organs, thereby contributing to favorable adaptive changes in tissues and systemic alterations [1,7]. These changes enhance the body’s physical performance and improve its overall functioning.

Organokines secreted during exercise are referred to as exerkines [3,8,9]. Exerkines influence human health in multiple aspects. They improve the functioning of the cardiovascular system, support metabolism, regulate immune functions, and affect neurological processes, including cognition, perception, and mood. They also show therapeutic potential in a variety of diseases, such as metabolic disorders, including type 2 diabetes, obesity, atherosclerosis, non-alcoholic fatty liver disease (NAFLD), referred to as metabolic-associated fatty liver disease (MAFLD), and chronic kidney disease (CKD). They also help slow the aging process and sarcopenia, positively influence cognitive processes, and delay/mitigate the progression of neurodegenerative diseases (Alzheimer’s disease, Parkinson’s disease, and others).

Contemporary studies suggest that most tissues are capable of communicating with others, both locally and at a distance. The crosstalk between tissues and organs is increasingly recognized as a crucial element in maintaining homeostasis and adapting to physical exercise or disease states. Many proteins with communicative functions remain undiscovered. Nevertheless, the known proteins and their function deepen the understanding of the crosstalk between the organs and may open new therapeutic avenues [10].

## 2. Irisin

Irisin is a polypeptide hormone discovered in 2012, secreted by skeletal muscles during physical exercise [11]. Structurally, it is a peptide molecule composed of 112 amino acids and belongs to the extracellular domain of the fibronectin type III domain-containing protein 5 (FNDC5), a membrane protein consisting of a 29-amino-acid signal peptide, a 94-amino-acid domain, and a C-terminal segment, which is believed to be the site of proteolytic cleavage before its release into the circulation as irisin [11,12,13,14,15]. In humans, this protein is encoded by the *FNDC5* gene, located on chromosome 1 at position 35.1 (1p35.1) [12]. Irisin is generated as a result of proteolytic cleavage from its precursor FNDC5, induced by PGC-1α (peroxisome proliferator-activated receptor gamma coactivator 1-alpha), one of the primary regulators of cellular energy metabolism [16]. Boström et al. [11], estimating the molecular mass of irisin post-cleavage from fibronectin type III, confirmed that the irisin in mice, rats, and humans is 100% identical. The level of irisin in the bloodstream is consistent with levels typical of polypeptide hormones, ranging from 3 to 5 ng/mL. It has been confirmed that physical exercise increases the circulating level of irisin in both humans and mice. In sedentary individuals, blood irisin levels average around ∼3.6 ng/mL, whereas, in physically active individuals, the levels can reach ∼4.3 ng/mL [17].

Initially identified in skeletal muscle, irisin was classified as a myokine. Subsequent studies have detected its expression in adipose tissue as well [18,19,20]. Boström et al. [11] suggested that irisin primarily functions in the activation of the browning process of white adipose tissue (WAT), contributing to increased energy expenditure. It plays a crucial role as a regulator of energy metabolism, as it is involved in glucose and free fatty acid oxidation processes, and acts as a modulator of brain-derived neurotrophic factor (BDNF), a neuroprotective agent [21]. Though present in smaller quantities, irisin has been detected in nearly all organs in both humans and rodents. It is found in the liver, pancreas, stomach, brain, spleen, heart, skin, retina, ovaries, and testes [12,20,22,23]. However, irisin expression varies significantly across tissues, with the highest levels observed in skeletal muscles [23]. By comparison, its expression in adipose tissue and the liver is approximately one hundred to two hundred times lower than in muscles [24]. Irisin has also been identified in cerebrospinal fluid [25], serum, saliva, urine [26], and milk [12,23].

Multiple factors influence the secretion of irisin, including physical activity, diet, pharmacotherapy, and pathological conditions. Among these, physical exercise appears to be the most potent stimulator of irisin production and secretion [23,27].

### Irisin—Molecular Mechanisms of Action

Skeletal muscles, regarded as endocrine organs, secrete irisin into the bloodstream in response to contractions triggered by physical exercise and the accompanying hypoxia. It has been demonstrated that irisin acts as a signaling molecule in skeletal muscles, the liver, adipose tissue, the heart, brain, and blood vessels [28]. According to Wojtaszewski et al. [29], exercise-induced hypoxia leads to a decrease in intracellular ATP levels and an increase in AMP (↓ATP/↑AMP). Elevated AMP levels stimulate AMPK (AMP-activated protein kinase). It has been confirmed that reactive oxygen species generated during aerobic metabolism also influence AMPK, which, in turn, stimulates the secretion of PGC-1α. This mechanism is activated as a result of disturbed resting homeostasis due to physical exercise and a shift in the redox balance, favoring the production of reactive oxygen species [30].

The AMPK→PGC-1α→FNDC5 pathway is one of the most crucial mechanisms in irisin synthesis. During physical exercise, the cytoplasmic Ca^2+^ concentration in muscle fibers significantly increases due to contractions. This rise induces AMPK phosphorylation, which subsequently stimulates PGC-1α and regulates the transcription of genes, including *FNDC5* [31,32].

PGC-1α is a key regulator of energy processes and a transcription coactivator engaged during hypoxia or ATP depletion in cells during exercise. It plays a central role in regulating cellular energy metabolism and mitochondrial biogenesis, particularly stimulated by regular physical activity [33]. PGC-1α interacts with numerous transcription factors involved in various biological responses, including adaptive thermogenesis, neurogenesis, angiogenesis, and the plasticity of skeletal muscle fibers (transforming them into slow-twitch (ST) oxidative fibers). It also prevents muscle dystrophy and denervation [15,33,34]. PGC-1α expression is also observed in the brain and kidneys, with a lower expression in the liver and minimal expression in WAT [33]. PGC-1α regulates carbohydrate and lipid metabolism, making it significant in conditions such as obesity and type 2 diabetes, and, thus, an attractive pharmacological target for treating these disorders. Cold exposure strongly stimulates its synthesis and plays a role in activating UCP-1 (uncoupling protein-1), which is localized in the mitochondria, dissipating energy and converting white adipocytes into beige (brite) adipocytes, thereby increasing energy expenditure, thermogenesis, and glucose homeostasis [14,16,35]. This action powerfully stimulates *FNDC5* gene expression, enabling the release of irisin from skeletal muscles.

Due to their unique mechanism of action, namely, increasing the mass and/or activity of BAT and converting white adipocytes into brown-like cells, BAT and beige adipocytes are considered attractive therapeutic targets for obesity and type 2 diabetes. Studies indicate that the activity of BAT adipocytes during the prenatal period, and beige adipocytes in WAT, which emerge postnatally, is regulated by distinct processes [36]. Unfortunately, the development and formation of beige adipocytes in WAT are still poorly understood. However, current knowledge suggests that they arise either through the conversion of existing white adipocytes and/or differentiation of adipocyte progenitor cells (APCs) residing in WAT [37,38].

Raajendiran et al. [39] argue that APCs constitute a reserve cell pool that can activate during regenerative processes following tissue damage. These cells are capable of proliferating and differentiating into mature cells, playing a critical role in maintaining the health potential of adipose tissue. They are also involved in tissue regeneration and in mitigating emerging dysfunctions. The authors assert that dysfunctional adipose tissue is closely linked to the development of metabolic diseases, such as dyslipidemia and type 2 diabetes. Authors research reveals the existence of three distinct APC subtypes, which are distributed differently across various WAT depots and may underlie the impaired adipocyte function observed in type 2 diabetes.

Other authors claim that the activation of beige adipocyte biogenesis improves glucose tolerance and insulin sensitivity, reduces inflammation and fibrosis in WAT, and protects against hepatic steatosis [40]. Therefore, beige adipocyte APC biogenesis disruption promotes the development of these conditions, as well as insulin resistance [41,42]. Moreover, the concentration of transforming growth factor-β (TGF-β), a key promoter of tissue fibrosis, is significantly elevated in the circulation and adipose tissue of obese mice and humans. The exposure of adipocyte precursor cells to TGF-β strongly inhibits beige adipocyte differentiation and UCP1 expression [40].

It has been proven that irisin exerts its biological effects via the PGC-1α/irisin/BDNF pathway [43] and through surface receptors from the integrin family, which are present in cells throughout the body [35]. Irisin acts via αV integrins; however, the mechanistic understanding of how small polypeptides like irisin can signal through integrins is poor. In various cell types (adipocytes, osteocytes, osteoclasts, and astrocytes), the irisin receptor has been identified as integrin αV/β5 [44,45].

However, irisin may also act as a ligand for the integrins αVβ1 and α5β1, which are expressed on mesenchymal cells, thereby playing a crucial role in the metabolic remodeling of bone, skeletal muscle, and adipose tissue [46]. Research indicates that irisin binds to the αV/β5 integrin in a two-step process via heat shock protein HSP90α (eHSP90α), secreted by skeletal muscles during exercise [47].

During inflammatory states, irisin circulating in the blood may bind to endothelial αVβ5 and act as an adhesive substrate for lymphocytes by binding to αLβ2 and α4β7, thereby regulating barrier function and modulating lymphocyte migration [46]. Irisin also binds to αVβ5, αVβ1, and CD81 on APCs, participating in the de novo biogenesis of beige adipose tissue [48]. The resulting complex mediates the activation of the integrin–focal adhesion kinase (FAK) signaling pathway. Furthermore, Oguri et al. [48] report that the loss of CD81 expression causes diet-induced obesity, insulin resistance, and adipose tissue inflammation. CD81 may act as a signal sensor, regulating the proliferation of beige adipocyte progenitors and, consequently, whole-body energy homeostasis.

**Figure 1:**
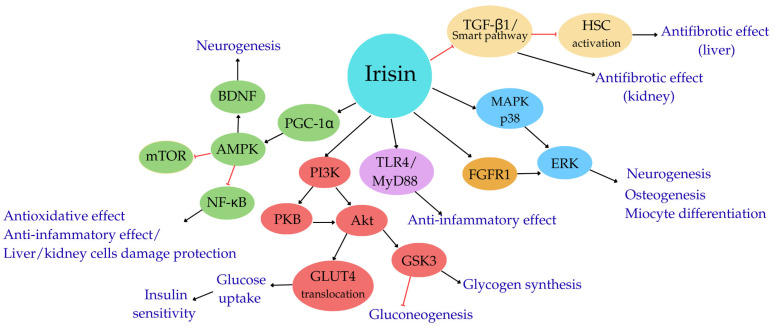
Intracellular pathways activated by irisin. Abbreviations used: BDNF—brain-derived neurotrophic factor; PGC-1α—Peroxisome proliferator-activated receptor gamma coactivator 1α; AMPK—AMP kinase; mTOR—mammalian target of rapamycin kinase, NFκB—Nuclear factor kappa-light-chain-enhancer of activated B cells; PI3K-PKB/Akt pathway—Phosphoinositide-3-kinase–protein kinase B/Akt; GSK3—Glycogen synthase kinase 3; GLUT-4—Glucose transporter type 4; TLR4/MyD88—Toll-like receptor 4 (TLR4)/Myeloid differentiation primary response 88; FGFR1—Fibroblast growth factor receptor 1; p38MAPK—p38 Mitogen-Activated Protein Kinases; ERK—Extracellular Signal-Regulated Kinase; TGF—β1-Transforming growth factor beta-1; HSC—hepatic stellate cells.

To better understand the biological actions of irisin, researchers are exploring numerous potential mechanisms of its protective effects via the activation of various cellular signaling pathways [49]. As mentioned, one such pathway involving irisin is the AMPK pathway (Figure 1). The enzyme plays a crucial role in regulating metabolic processes, acting as an energy sensor within cells and regulating energy flow by modulating glucose and lipid metabolism. Irisin activates the AMPK pathway by stimulating receptors on the surface of myocytes [50].

Studies have shown that irisin also increases the expression of PGC-1α, thereby activating the AMPK pathway. The activated AMPK inhibits the activity of the mammalian target of rapamycin (mTOR) pathway, which senses the cellular ATP levels and redox balance [11,51].

The nuclear factor kappa B (NF-κB) signaling pathway is another regulatory mechanism in which irisin participates. As a transcription factor present in all somatic cells, NF-κB plays a critical role in regulating inflammatory, immune, and apoptotic processes. Numerous stimuli, including cytokines, chemokines, reactive oxygen species, and other stress factors, activate the NF-κB pathway. Additionally, it is involved in processes related to synaptic plasticity, including cognitive functions and memory [52]. Through the NF-κB pathway, irisin exerts anti-inflammatory effects. Inhibiting NF-κB activity reduces the secretion of pro-inflammatory cytokines and dampens the inflammatory response. Furthermore, irisin may act as an antioxidant via this mechanism, protecting cells from oxidative injury [43].

Research by Mazur–Biały et al. [53] suggests that irisin exerts anti-inflammatory effects by stimulating the toll-like receptor 4/myeloid differentiation primary response 88 pathway (TLR4/MyD88) in macrophages, thereby supporting the human body’s defense against infections.

Irisin also affects the phosphatidylinositol 3-kinase/protein kinase B signaling pathway (PI3K/Akt), which is involved in regulating glucose metabolism, cell growth, and survival. It influences this pathway by activating membrane receptors and increasing the activity of protein kinase B (Akt). Irisin-activated Akt stimulates the translocation of glucose transporter type 4 (GLUT4) from intracellular compartments to the cell membrane in skeletal muscle cells, thereby enhancing glucose uptake from the blood. Additionally, the activation of the PI3K/Akt pathway by irisin stimulates protein anabolism and inhibits muscle protein catabolism, contributing to increased muscle mass [54].

According to Boström et al. [11], irisin may stimulate the differentiation of muscle cells by activating the mitogen-activated protein kinase/extracellular signal-regulated kinase pathway (MAPK/ERK). The transcription factors expressed as a result of this activation induce muscle repair and regeneration processes. Studies have shown high MAP kinase activity in the central nervous system. ERK kinases are involved in long-term neuronal plasticity, including long-term synaptic potentiation, which promotes memory consolidation [55]. Researchers have also demonstrated that irisin may activate the MAPK/ERK pathway through interaction with fibroblast growth factor receptor 1 (FGFR1), which is present on the surface of muscle cells. In mouse studies, irisin increased the expression of the myogenin gene, a key transcription factor regulating muscle cell differentiation [56].

Furthermore, Qiao et al. [57] suggest that irisin promotes the proliferation and differentiation of osteoblasts via the MAPK pathway. Similar conclusions were drawn by Kim et al. [45] and Rabiee et al. [58], who indicated that irisin may play a significant role in the treatment of metabolic and musculoskeletal disorders. However, further research is necessary in order to confirm these hypotheses and fully understand the role of irisin in regulating physiological processes in humans [58].

## 3. Irisin and Physical Exercise

Current findings confirm that irisin is secreted during physical activity or training; however, there is a lack of consensus regarding which type of exercise most significantly influences its expression. This inconsistency is likely due to the variability in assay methods, research methodologies, the selection of study groups, exercise type and duration, and the timing of the irisin measurement in the plasma or serum after exercise. Some studies report an increase in irisin concentration following individual training sessions, while others do not confirm this. The same applies to training intensity. Initially, it was believed that high-intensity training (HIIT) most strongly stimulated irisin concentration [59], but later studies either did not confirm this [60] or observed only a minimal increase [61]. Nevertheless, several authors affirm a strong correlation between irisin levels and physical training. Increases in irisin concentration have been observed following resistance training [62], HIIT [63], and endurance and aerobic training [11,17,60], as well as strength training [64]. Table 1 provides the details of the studies assessing irisin concentration in the blood following various types of physical activity/training.

Although the critical role of irisin in the human body is widely recognized, it is essential that we emphasize that the methods used to determine its concentration in human and animal blood raise specific concerns [65]. Most tests used to measure irisin levels in the blood are commercially available enzyme-linked immunosorbent assay (ELISA) kits, which have low specificity and sensitivity, and provide inconsistent results. A particular point of concern is that the human FNDC5 gene contains an atypical translation start codon (ATA instead of the classical ATG, as seen in mice and rats) [11,66]. Based on this, some authors believe that translation may not occur in humans at all.

Commercially available ELISA kits also exhibit significant discrepancies in the results obtained from identical samples [66]. Since the tests use polyclonal antibodies, frequent cross-reactivity with other proteins significantly reduces the reliability of the results [67]. Another critical element in the analytical process is the preliminary step of preparing serum/plasma samples to minimize the pre-analytical error. Due to the existence of numerous ELISA kit manufacturers, reference values for irisin concentrations in serum/plasma under resting conditions, post-exercise [68], and in various pathological states are currently lacking. Therefore, irisin is not a reliable marker of physiological changes, particularly in clinical research. Nonetheless, many studies propose irisin as a post-exercise marker or as an indicator of disease progression or recovery following rehabilitation.

Huh et al. [50] confirmed that irisin levels positively correlate with training intensity. The authors suggest that the exercise-induced surge in irisin likely originates predominantly from the skeletal muscle release. Moreover, although the irisin level change induced by exercise is approximately 10–30%, the local concentrations within skeletal muscles during training may be significantly higher than those observed in circulation.

Boström et al. [11], in studies conducted on mice and human skeletal muscle biopsies, demonstrated that irisin concentration increases with physical activity. Their findings indicated that plasma irisin levels in mice rose by 65% after three weeks of voluntary wheel running, while, in healthy humans, the levels doubled after a 10-week endurance training program compared to non-exercising controls.

Murawska-Ciałowicz et al. [63] also confirmed an increase in irisin levels after 8 weeks of training in young, minimally active men. Hew-Butler et al. [60] observed that blood irisin levels may depend on the intensity, the type of exercise performed, and the degree of physiological adaptation. As expected, irisin concentration correlates positively with physical activity and muscle mass; hence, it is higher in physically active individuals compared to less active or sedentary individuals.

Other researchers emphasize a positive correlation between age-related muscle mass loss (sarcopenia) and blood irisin levels [50]. Löffler et al. [69] showed that the irisin concentration declines with age. In adults, this hormone’s level was higher in men than in women and higher in obese individuals compared to lean individuals. Additionally, they found that irisin concentration was strongly correlated with lean body mass [69].

It is believed that the factors influencing FNDC5 levels and, indirectly, irisin levels include low ambient temperature, physical activity, and leptin concentration, which increase muscle mass [34]. Considering the beneficial health effects of physical activity on energy metabolism, cardiovascular function, obesity, diabetes, and skeletal disorders, the authors’ view that irisin could serve as a potential therapeutic agent for metabolic and lifestyle-related diseases is well-justified [28,34].

According to Lagzdina et al. [70], the total pre-exercise irisin levels were similar in men and women but higher in women when normalized for body weight. In men, there was an inverse correlation between pre-exercise irisin levels and visceral fat content, and a positive correlation with the lean body mass (LBM) and resting metabolic rate in both sexes. Furthermore, 58% of participants showed no significant change in irisin concentration post-exercise, 23% exhibited a decrease, and 19% an increase.

Irisin secretion is influenced by sex and the circadian rhythm. The lowest levels are observed at 6:00 AM and the highest at 9:00 PM [71]. According to the same authors, irisin concentration positively correlates with lean body mass and the female sex.

The multitude of variables affecting irisin secretion in response to physical activity necessitates further research in order to clarify the regulation of this hormone’s release. Understanding these mechanisms is crucial for elucidating irisin’s diverse effects in the context of physical performance and its potential protective role in various pathological states, especially since the latest research indicates the important role of physical exercise and skeletal muscles in the regulation of metabolism and the pathogenesis of chronic non-communicable diseases.

## 4. Liver and Physical Exercise

Earlier studies suggested that physical activity is not an effective therapeutic method in the context of general liver failure, as it leads to an increase in hepatic metabolism. Reduced physical activity lowers the liver’s metabolic load, thereby reducing the risk of complications [72]. However, more recent studies have demonstrated that prolonged inactivity can lead to muscle atrophy [73], deep vein thrombosis [74], and an increased risk of liver fibrosis. Consequently, the initial approach has been revised. Physical exercise has proven effective in treating complications of liver cirrhosis and in reducing the risk of liver cancer. Regular physical activity improves the quality of life in patients with liver diseases and post-transplant individuals, and it alleviates metabolic disorders such as obesity and diabetes [75].

Physical activity primarily includes aerobic and resistance training, each offering distinct benefits and indications in the context of liver disorders. Aerobic training effectively reduces body weight, glycated hemoglobin (HbA1c) levels, blood pressure, and cholesterol concentration in the blood. Resistance training, which involves muscle contractions in response to external resistance to enhance strength, bone density, and endurance, provides significant benefits in the treatment of dyslipidemia, hypertension, and insulin resistance. It yields metabolic effects with a lower energy expenditure [76,77,78].

To meet the accelerated metabolic demands of working muscles, intensive hepatic activity is essential. Without it, prolonged physical exertion would be impossible. The liver plays a pivotal role in storing, releasing, and processing energy substrates. Without an accelerated glucose release in response to exertion, hypoglycemia could occur. The liver’s increased energy demand is met by the enhanced oxidation of fatty acids mobilized from adipose tissue. Post-exercise regenerative processes support the replenishment of glycogen stores, which serve as a glucose source for active muscles. The pancreatic hormones, glucagon and insulin, coordinate hepatic responses both during and after physical activity. Like skeletal muscles and other body systems, the liver adapts to repeated exercise stress, enhancing its capacity to generate energy through fat oxidation. Regular physical activity promotes this adaptation, protecting the liver and potentially reversing hepatic steatosis, thereby improving its metabolic performance [79].

During intense exertion, hepatic glycogenolysis accelerates along with the energetically costly process of gluconeogenesis to supply glucose to working muscles. Exercise induces the adaptation of hepatic glucose and lipid metabolic pathways to more effectively meet ATP demands [80]. Studies by Hughey et al. [80,81] in mice confirmed that AMPK, functioning as a cellular energy sensor and regulator, plays a key role in several exercise-stimulated mechanisms. It is crucial for regulating glycogen and lipid availability, and necessary for increasing hepatic glycogen content, which enables accelerated glycogenolysis during exercise. AMPK also contributes to the reduction in harmful hepatic glycerolipids and selectively alters the fatty acid chain composition in diacylglycerol molecules in response to exercise, which may be significant for proper liver function and overall energy metabolism.

## 5. Irisin–Liver–Physical Exercise

The liver is one of the target organs for the physiological effects of irisin, as irisin regulates blood glucose levels by controlling glucose production, uptake, storage, and release through glycogen synthesis and degradation [23].

Irisin facilitates glucose uptake by skeletal muscles, increases the expression of glucose transporters (GLUT-4) on muscle fiber surfaces, enabling glucose influx into them, improves glucose and lipid metabolism in the liver, and regulates hyperlipidemia and hyperglycemia caused by obesity and metabolic syndrome. Thus, it acts as an insulin-sensitizing hormone. Accordingly, irisin may exert positive effects on the liver and pancreatic islets, thereby reducing the risk of type 2 diabetes [18,34]. Irisin levels have also been found to be positively correlated with metabolic syndrome parameters: systolic blood pressure, fasting glucose levels, and triglyceride levels [15].

In hepatocytes, irisin inhibits gluconeogenesis, lipogenesis, and lipid accumulation, as illustrated in Figure 1 [32,82].

Liu et al. [82] report that insulin-resistant hepatocytes treated with irisin show reduced gluconeogenesis and increased glycogen synthesis. Irisin exerts similar effects in the livers of obese mice. According to Tang et al. [83], irisin downregulates genes involved in cholesterol synthesis while upregulating genes responsible for cholesterol metabolism via AMPK action, ultimately reducing the plasma and hepatic cholesterol content in diet-induced obese mice. In these models, recombinant irisin injections over two weeks inhibited hepatic cholesterol synthesis through AMPK activation.

Studies by Mo et al. [84] showed that irisin inhibits lipogenesis and improves cholesterol metabolism in hepatic steatosis in mice.

Hepatic lipid metabolism plays an equally important role in glucose homeostasis. When the carbohydrate intake exceeds the storage and oxidation capacity, they are converted into lipids via de novo lipogenesis [34]. However, excessive hepatic lipid levels lead to inflammation and fatty liver. Hepatic stress, induced by lipid overload, alcohol, psychological stress, and disrupted redox processes, negatively affects liver health [85]. Park et al. [86] demonstrated that recombinant irisin reduces exercise-induced hepatic lipogenesis and excessive lipid accumulation.

The activation of hepatic AMPK has been shown to exert antidiabetic effects by modulating glucose and fat metabolism, inhibiting lipogenesis and gluconeogenesis, and promoting lipid oxidation and glycolysis [34,83]. Irisin expression was significantly reduced in patients with non-alcoholic steatohepatitis (NASH) and in murine models of liver injury induced by ischemia-reperfusion (I/R) [32]. Studies by Bi et al. [87] found that treatment with exogenous irisin (250 μg/kg) promoted liver function, reduced hepatocyte necrosis and apoptosis, and alleviated inflammation following hepatic I/R injury.

Irisin reduces reactive oxygen species (ROS) through its antioxidant activity. Disrupted redox homeostasis is one of the factors leading to structural and functional liver damage. Irisin’s antioxidant mechanism includes both reducing ROS levels and mitigating their damaging effects. It regulates key cellular processes such as mitochondrial division and autophagy, thereby limiting ROS and oxidative stress [85].

Many researchers report that oxidative stress is one of the primary mechanisms underlying liver disease development, regardless of etiology, including NAFLD, ischemia-reperfusion injury, or cirrhosis [85]. Irisin, therefore, acts as a modulator of inflammation and oxidative stress in liver injury [32]. According to Bi et al. [87], irisin mitigates liver damage resulting from ischemia-reperfusion by promoting mitochondrial biogenesis and reducing oxidative stress.

The endoplasmic reticulum (ER) is an essential organelle that controls protein translation [88]. The ER responds to cellular disturbances disrupting normal function and is involved in a signaling pathway known as ER stress. It has been confirmed that ER stress is positively correlated with ROS production. Irisin exerts a sustained influence on hepatic cell function under oxidative and ER stress, suggesting that ER stress is at least partially associated with irisin’s antioxidant mechanism [85,89]. Research by Bi et al. [87] highlights the beneficial/therapeutic effect of irisin in ischemia/reperfusion-induced injury in both lungs and liver.

Recent studies confirm irisin’s anti-inflammatory properties, demonstrating its ability to inhibit inflammatory responses in various cell types, including adipocytes, macrophages, hepatocytes, pancreatic β-cells, and endothelial cells. This mechanism is based on the NF-κB and NOD-like receptor family, pyrin domain containing 3 (NLRP3). These findings suggest that irisin may function as a potent immunometabolic regulator in the treatment of obesity and related metabolic diseases [4,90].

According to Canivet et al. [91], *FNDC5* expression increased in the liver due to hepatic steatosis and liver damage. However, this did not affect the irisin concentration in the blood in murine NAFLD models or obese patients. An increased irisin expression was primarily observed in hepatocytes and non-parenchymal cells. According to the authors, this makes irisin a potent protective factor that reduces the symptoms of hepatocyte fibrosis. Using a real-time polymerase chain reaction (RT-PCR), the authors demonstrated a very low *FNDC5* expression in endothelial and Kupffer cells compared to hepatocytes, and an over fourfold higher expression in isolated hepatic stellate cells (HSCs) compared to hepatocytes. The authors suggest that, due to the proportion of hepatocytes relative to non-parenchymal cells (~70–80% hepatocytes: 20–30% non-parenchymal cells), hepatocytes may play a key role in irisin expression.

However, HSCs are primarily responsible for the development and pathogenesis of liver fibrosis [92,93,94]. These cells are in direct contact with nerve endings, which supports their role in neuro-humoral interactions [94]. In response to damaging factors, HSCs become activated and acquire myofibroblastic features, synthesizing large quantities of collagen type I [95,96]. HSC activation is accompanied by the activation and infiltration of Kupffer cells, which secrete ROS and various pro-inflammatory cytokines, including TGF-β1, thereby stimulating cellular proliferation and extracellular matrix synthesis by HSCs.

In a study by Dong et al. [97] on HSCs, recombinant irisin significantly inhibited the expression of fibrosis markers stimulated by TGF-β1, such as smooth muscle α-actin and collagen type I alpha 1, and prevented the TGF-β1-induced proliferation, migration, and contractility of the cells. Integrins, which are present in all nucleated cells, also play an important role in the pathomechanisms of liver fibrosis. The regulatory effect of integrins on TGF-β activity appears to involve mainly integrins containing the αv subunit, which selectively bind to the Arg-Gly-Asp motif, activating TGF-β. Indeed, αvβ6 and αvβ8 show the highest affinity for TGF-β. The expression of αvβ1, αvβ3, αvβ5, and αvβ8 has been demonstrated in activated HSCs, while endothelial cells display αvβ3 and αvβ5.

Integrins are transmembrane proteins that constitute a family of cell surface receptors that are the primary receptors involved in cell adhesion. They are heterodimeric, composed of α and β subunits, which results in many types of integrins with diverse ligand affinities. By binding to ligands such as irisin, collagen, and fibronectin, among others, in the extracellular matrix, integrins initiate intracellular signaling pathways that regulate various cellular processes [98]. They participate in organ development, tissue repair, and inflammatory responses, and their dysfunction is associated with various diseases [99], including heart failure [100]. They are also involved in synaptic plasticity [101] and play a role in lung development, regeneration, and inflammation. A key function of integrins is their role in angiogenesis [99], which is crucial in tissue repair and improving organ perfusion [102].

In studies by Lai et al. [103], irisin attenuated HSC activation, alleviated liver fibrosis, and improved the associated mitochondrial dysfunction. These findings highlight the therapeutic potential of irisin in the treatment of liver fibrosis.

The development of systemic inflammation, as well as functional and filtration disturbances of the liver caused by various factors, and a low level of physical activity, promotes the onset of numerous chronic liver diseases (CLD), including NAFLD, liver fibrosis, cirrhosis, and hepatic cancers. Liver fibrosis occurs in all CLDs, including NAFLD.

The global prevalence of NAFLD is 25–30% [104], and it is an increasingly common health issue in modern societies. Projections estimate that, by 2030, the NAFLD prevalence will increase by 21% (approximately 100 million) in the United States alone, and NASH will increase by 63% (to 27 million) [105].

NAFLD is a significant public health concern in developed societies due to its high prevalence, potential for progression to severe liver disease, and association with major cardiovascular and metabolic disorders. Indeed, NAFLD is linked to a high risk of developing diabetes, dyslipidemia, and hypertension [106]. According to Fabrinni et al. [106], the development of NAFLD and its progression to cirrhosis and hepatocellular carcinoma is partly due to insufficient physical activity.

According to Croci et al. [107], a sedentary lifestyle increases the risk of NAFLD by as much as 4%. Lifestyle modification, particularly dietary changes and increased physical activity, remains the cornerstone of preventing and treating this metabolic disease.

According to Chalasani et al. [108], a weight reduction of 5–10% is considered sufficient for achieving histopathological improvement in NASH. However, the authors note that fewer than 10% of patients achieve the desired degree of weight loss, and more than 75% of those patients return to their baseline weight within approximately three years of discontinuing physical activity.

Physical activity promotes the reversal of histological changes associated with NASH, reduces the hepatic fat content, and restores impaired cellular pathways through various cellular mechanisms [108,109].

It is believed that, in combination with a moderate weight loss of at least 7%, regular physical activity may even improve the status of fibrotic tissue in the liver. Liver fibrosis can be a consequence of metabolic disorders, including diabetes, overweight, obesity, and steatohepatitis [110]. Studies by Pang et al. [111] also suggest that physical activity can reduce the risk of developing liver cancer.

The “Exercise as Medicine” program developed by the American College of Sports Medicine recommends that patients with chronic liver disease engage in moderate-intensity physical activity for at least 150 min per week. They also advise body-weight resistance training (calisthenics) twice per week to increase muscle mass [112]. Similar dietary and physical activity recommendations are presented for NAFLD patients in the study by Glass et al. [113].

According to Stine et al. [114], physical training in patients with NAFLD resulted in a 3.5-fold reduction in visceral fat compared to those receiving standard clinical care.

In the liver, physical activity increases the oxidation of fatty acids and glucose, enhancing their utilization to meet the body’s demands during exercise, reduces fatty acid synthesis, and prevents mitochondrial damage. Anti-inflammatory effects represent just part of the beneficial biochemical and cellular outcomes [115]. Based on the cited studies, there is substantial evidence indicating that physical activity enhances liver function by activating numerous mechanisms.

Yazdani et al. [116] believe that these beneficial effects are related to reduced oxidative stress, the scavenging of free radicals (such as ROS), a reduction in inflammation, the regulation of M1/M2 macrophage polarization, increased β-oxidation, and decreased intrahepatic fat content.

Physical training may induce hepatic irisin expression, inhibit inflammatory responses, and improve liver function in NAFLD by activating toll-like receptor 4 (TLR4) [117]. According to recommendations presented by Kistler et al. [109] regarding physical activity in patients with NAFLD, high-intensity exercise is preferred as it provides the most favorable effects on liver function. The authors argue that intensity is more critical than duration or total exercise volume in improving liver function.

Hew-Butler et al. [60] also found that blood irisin levels depend on exercise intensity, type of physical activity, and the degree of physiological adaptation. Irisin levels positively correlate with physical activity and muscle mass, which is why physically active individuals have higher irisin concentrations compared to those who are less active or sedentary.

In a study by Tine Kartinah et al. [118], rats underwent HIIT by running on a treadmill five times per week for eight weeks, with the speed increasing weekly. The authors observed that irisin expression was significantly higher in the HIIT group than in the continuous moderate-intensity training (CMIT) group. They concluded that HIIT induces greater physiological stress, thereby stimulating greater irisin secretion.

Wang et al. [119] studied the effects of aerobic exercise in rats with post-myocardial infarction liver injury and confirmed that exercise increased FNDC5/irisin expression and activated the PI3K/Akt signaling pathway, thereby inhibiting hepatic inflammatory responses.

Belviranlı and Okudan [120] indicate that regular exercise inhibits the age-related decline in irisin levels in the heart, liver, and plasma. Irisin concentrations in these organs and plasma were lower in sedentary older rats compared to both sedentary young rats and exercising young and old rats. Regular exercise increased irisin levels in all analyzed tissues compared to sedentary animals.

Recent findings indicate that the crosstalk between organs is critical for energy homeostasis. Previous research suggests direct signaling between skeletal muscle and the liver via the irisin protein. Tang et al. [83] provided significant evidence that hepatocytes are targets for irisin in the context of hepatic cholesterol metabolism regulation. The role of the irisin receptor in the liver has not yet been clearly defined, and the question of whether irisin first activates surface receptors on hepatocytes before regulating glucose and lipid metabolism remains unanswered [32].

**Figure 2:**
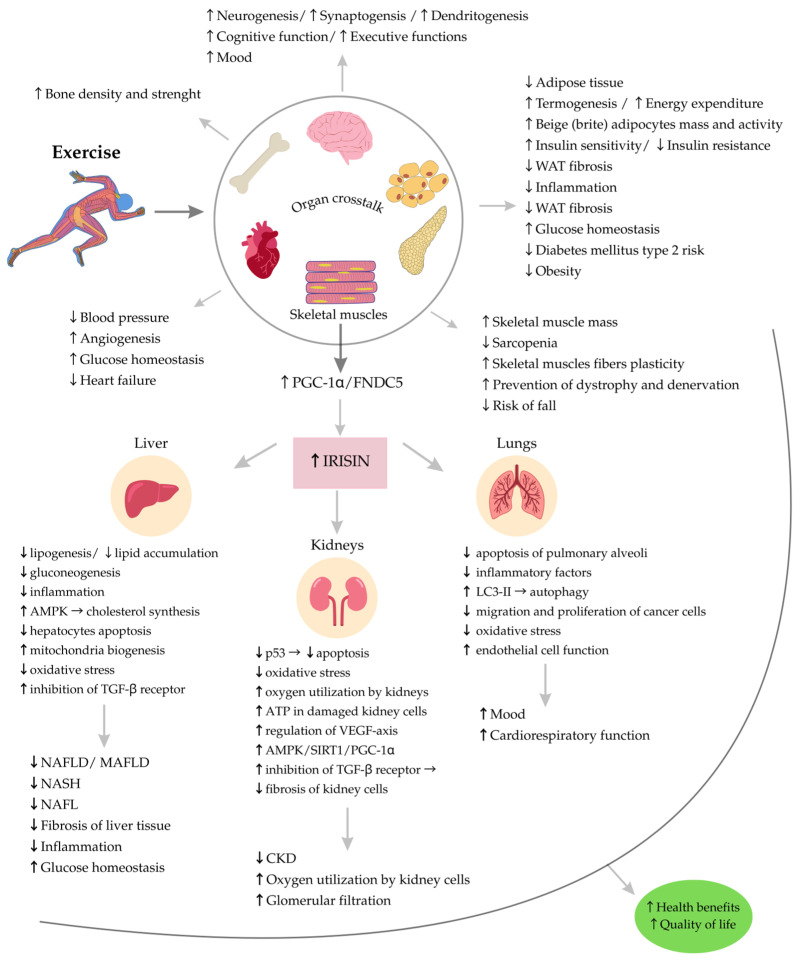
The effect of irisin on protective mechanisms in various organs, with particular emphasis on the liver, kidneys, and lungs. ↓—lower/decrease; ↑—higher/increase/activation.

Collectively, the available data indicate that irisin plays a significant role in regulating liver function by positively influencing glucose and lipid metabolism, reducing inflammation and oxidative stress. This hormone may enhance insulin sensitivity and protect the liver from damage associated with obesity, metabolic syndrome, and other stressors. Research findings also suggest that irisin could be a potential therapeutic target in the treatment of metabolic and liver diseases. Despite extensive experimental evidence, further research is required in order to fully understand irisin’s mechanisms of action in hepatocytes, particularly regarding its receptor and intracellular signaling pathways. The potential mechanisms of irisin action in the liver and other organs are presented in Figure 2.

Moreover, for irisin to be recognized as a significant biomarker in liver diseases, including NAFLD, there is a need for a greater number of clinical studies and the development of accurate and specific laboratory tests that would enable the diagnosis of liver diseases based on changes in irisin concentration. Research in this area remains limited, and some of these studies are shown in Table 2.

A meta-analysis by Hu et al. [128] found no differences in irisin concentration between healthy individuals and patients with NAFLD. In contrast, a meta-analysis by Shen et al. [129] showed that circulating irisin levels were significantly lower in patients with MAFLD compared to healthy individuals. The authors emphasize that different analytical tests were used in the studies, which may have influenced the results. Therefore, there is a need to standardize the methodology for irisin measurement to ensure a reliable diagnosis based on this biomarker.

## 6. Kidneys and Physical Exercise

According to studies by Poortmans [130,131], physical exercise induces profound changes in renal hemodynamics as well as in the excretion of electrolytes and proteins. Exercise leads to a decrease in renal blood flow (RBF), which is proportional to the intensity of the activity. RBF can decline significantly, even by up to 25% compared to resting values. During physical exertion, there is also a reduction in effective renal plasma flow (RPF) and in the glomerular filtration rate (GFR). Existing research shows that these indicators are more dependent on exercise intensity than duration. Poortmans [130] observed no changes in GFR during low-to-moderate-intensity exercise. Similarly, Suzuki et al. [132] found that creatinine clearance, a key marker for assessing GFR, begins to gradually decline only at exercise intensities between 42.5% and 60.5% of VO_2_max. However, it dropped immediately to 47% and 45% when the exercise intensity reached 83% and 100% of VO_2_max, respectively. Suzuki et al. [132] also reported that, during the recovery period following exhaustive cycling exercise, RPF decreased to 46.6% compared to pre-exercise values, and during restitution, returned to 82.5% at 30 min and 78.9% at 60 min post-exercise. The authors attributed these changes to increased sympathetic nervous system activity in the kidneys and heightened catecholamine secretion during both exertion and recovery [132,133]. The renin–angiotensin–aldosterone (RAA) system also plays a regulatory role [133].

Furthermore, it has been shown that proteinuria differs post-exercise compared to resting conditions and is dependent on exercise intensity [134]. A glomerulotubular type of proteinuria predominates after high-intensity exercise, whereas a glomerular type is typically observed following low-intensity exercise. The clearance of specific plasma proteins during exercise suggests an increased glomerular permeability and the partial inhibition of the tubular reabsorption of macromolecules [131].

During high-intensity exercise, a significant effect of the antidiuretic hormone (vasopressin) is also observed, as the filtration rate depends on the level of body hydration. Additionally, it has been found that intense, single-session physical effort can lead to the apoptosis of cells in the distal renal tubules. This is likely associated with a free-radical mechanism and the involvement of angiotensin II receptors AT1 and AT2 [135]. Protein excretion in urine is a temporary condition and can persist for up to two hours post-exercise [136]. Hemoglobinuria and myoglobinuria are also observed following exertion. Myoglobin serves as a marker of rhabdomyolysis (skeletal muscle damage due to physical effort) [132].

Contemporary perspectives on the effects and role of exercise in kidney function differ from the earlier belief that physical activity is harmful, particularly in individuals with renal impairment. Current studies indicate that physical activity reduces the risk of kidney disease, delays the progression of CKD, and is recommended in the prevention and treatment of conditions accompanied by kidney failure [137]. Some research highlights the health benefits of physical activity in dialysis patients. However, not all forms of exercise are suitable for patients with kidney disease, as they often suffer from comorbidities such as cardiopulmonary insufficiency, diabetes, hypertension, or depression. Therefore, moderate aerobic activities such as walking, swimming, and cycling are recommended [138]. High-intensity anaerobic exercise is discouraged, as it causes profound changes in renal hemodynamics and the excretion of electrolytes and proteins [132,134,136]. For patients with mild renal insufficiency, daily physical activity at an intensity of 35–40% VO_2_max is advised, as hemodynamic parameters reflecting kidney function do not deteriorate during exercise within this intensity range [139]. This level of effort generally corresponds to intensity below the lactate threshold. Fukuta et al. [140] demonstrated that, in healthy individuals, RBF below the ventilatory threshold does not differ from resting values but begins to decline significantly only once the threshold is exceeded.

## 7. Irisin–Kidneys–Physical Exercise

Some authors indicate that both aerobic and resistance training bring significant health benefits related to kidney function. Aerobic exercise improves overall functional capacity, while resistance training increases strength, power, and muscle mass. Experimental studies in rats have also shown that physical activity contributes to the reduction in inflammatory processes and the progression of renal fibrosis [141]. Moreover, in experimental models of kidney disease, aerobic exercise has been observed to reduce inflammation and the apoptosis of kidney cells [142].

Recent studies increasingly emphasize the importance of signaling between different systems and organs. This signaling can either exacerbate pathological changes or provide protective effects on other tissues, even in the absence of direct functional connections. An example of this is the correlation between kidneys and CKD [143]. A strong association has been demonstrated between sarcopenia and renal dysfunction in patients with CKD. Conversely, it has also been proven that substances produced and secreted by the kidneys can impair muscle function [143,144,145].

Knowledge of the crosstalk between organs is used in the prevention and treatment of various diseases through physical activity and the enhanced expression/synthesis of myokines in skeletal muscle. This understanding may be applied therapeutically as a regulatory mechanism in conditions such as cachexia and sarcopenia, commonly observed in CKD patients and those undergoing dialysis, as well as in various physiological processes, including aging. It has been established that such a communication system exists between skeletal muscles and the kidneys, and that myokines secreted during exercise participate in regulating kidney function. Specifically, irisin has been identified as a myokine that communicates between skeletal muscles and the kidneys, exerting a protective effect on the latter [145].

Despite the significance of this phenomenon, few studies examine the correlation between physical activity and myokines, including irisin, in the context of kidney function regulation in healthy individuals or athletes [137]. Similarly, there is a lack of studies conducted in animal models or cell cultures. Most research on irisin’s role in the kidneys primarily concerns CKD.

CKD is a condition characterized by multiple physiological disturbances, including metabolic acidosis, systemic inflammation, and increased oxidative stress, which promote the development of sarcopenia [146,147]. However, the mechanisms explaining CKD’s impact on muscles and bones remain poorly understood. Leal et al. [146] suggest a crosstalk between muscle and bone tissue, indicating that impaired myokine synthesis can influence them.

CKD causes a mineral imbalance in bones and a decrease in bone mineral density (BMD). It is also associated with reduced vitamin D levels, secondary hyperparathyroidism, and sarcopenia. Studies by Kawao and Kaji [148] further show that sarcopenia coexists with the disrupted bone metabolism. The effect of muscle on bone metabolism results from the secretion of several myokines, including irisin, follistatin, insulin-like growth factor-1 (IGF-1), transforming growth factor-beta (TGF-β), myostatin, and fibroblast growth factor 23 (FGF23), which interact to influence the bone tissue condition.

In healthy individuals, muscle mass is maintained through a balance between anabolic and catabolic processes [149], namely, the balance between the IGF-1/Akt pathway, which induces protein synthesis and satellite cell recruitment in muscles, and the opposing myostatin pathway, which promotes protein breakdown and inhibits satellite cell recruitment [150]. Muscle loss results from an imbalance between these two systems [149,151]. Studies have shown higher levels of myostatin in patients with CKD and those undergoing dialysis compared to healthy individuals. In CKD, an increased myostatin production is primarily associated with low levels of physical activity, inflammation, oxidative stress, uremic toxins, metabolic acidosis, and angiotensin II [149].

Myostatin is the primary regulator of muscle development. Once released from muscles, it can act locally and systemically through activin type IIB receptors, which form a complex with TGF-βRI and induce intracellular signaling via p38MAPK and NFκB [152,153]. In skeletal muscle cells, myostatin acts as an inhibitor of myocyte hypertrophy and exerts endocrine effects on other organs, including bone metabolism [153]. Myostatin increases the level of sclerostin in osteocytes, which inhibits bone formation [154]. Thus, myostatin participates in the crosstalk between the muscular and skeletal systems. Chronic and short-term physical exercise both decrease myostatin levels in muscle tissue and blood serum [155].

Additionally, reduced myostatin levels decrease fat content and improve glucose metabolism [156]. In a study by Li et al. [157], elevated myostatin levels and reduced irisin concentrations were observed in patients with sarcopenia. According to the authors, increased myostatin levels are a risk factor for sarcopenia, while irisin levels serve as a protective factor. Both substances are considered markers of strength and physical fitness in older patients [158]. Follistatin binds to myostatin, inhibiting its activity. In mouse models, follistatin has been shown to increase the skeletal muscle mass significantly [156,159].

Ewend et al. [152] demonstrated that myostatin regulates the production of FGF23, a paracrine and endocrine mediator produced by bone cells that regulates phosphate and vitamin D metabolism in the kidneys. FGF23 is also a hormone that increases urinary phosphate excretion and inhibits the production of 1,25-dihydroxyvitamin D in the kidneys. In this way, it contributes to alleviating hyperphosphatemia in patients with kidney diseases. Hyperphosphatemia and low levels of 1,25-dihydroxyvitamin D are associated with mortality in patients with CKD. FGF23 is also considered a marker of inflammation and an independent prognostic marker of mortality in patients with CKD [160,161,162,163].

FGF23 may also play a pro-inflammatory role in chronic obstructive pulmonary disease (COPD), as elevated levels of this hormone have been demonstrated in COPD patients compared to a control group [164,165].

Kawao and Kaji [148] demonstrated that kidney failure suppresses irisin expression in muscles, which otherwise limits bone loss by acting on bone tissue. The effects of CKD on muscle–bone interactions remain unclear [148]. Irisin appears to enhance osteocyte survival via integrin αV/β5 receptors [45,166]. Studies show that the mechanical load from resistance exercise is transmitted from skeletal muscle to bone tissue, initiating both muscle protein synthesis and signaling energy demands to facilitate bone formation, providing evidence of this crosstalk [16].

There are few clinical studies describing changes in irisin concentrations in CKD. A review of the limited research in this area, linking irisin to CKD, is shown in Table 3.

CKD patients typically exhibit lower serum irisin levels, suggesting that low irisin levels may constitute a risk factor for CKD [167,168,169].

In studies on mice, renal failure was shown to suppress irisin expression in muscle, while irisin reduced bone tissue loss [171]. Shelbaya et al. [170] also found decreased serum irisin levels in patients with type 2 diabetes and diabetic kidney disease. These findings underscore irisin’s potential relevance in pathological kidney states.

Rodrigues et al. [172] demonstrated the nephroprotective effects of aerobic exercise in experimental diabetic nephropathy, though, at the time, the molecular mechanisms were not well-understood. Formigari et al. [173] observed that aerobic exercise in diabetic rats reduced albuminuria, glomerular hypertrophy, and the expression of collagen IV and fibronectin, as well as inflammatory markers in renal glomeruli. These effects were attributed to increased muscle irisin levels and AMPK activity. These beneficial changes were reversed by the administration of Cyclo (RGDDyK), an irisin pathway inhibitor. Similarly, recombinant irisin administered to cultured human kidney tubular cells (HK-2) activated AMPK and showed positive effects. It is thus inferred that, in diabetes, irisin-mediated AMPK activation may explain the renal protective effects of physical exercise.

Liu et al. [174] observed irisin induction in renal tubular cells in a mouse ischemia/reperfusion (I/R) model and cultured mouse proximal tubular cells under ATP depletion. Silencing the FNDC5 gene increased ATP-depletion-induced apoptosis, whereas FNDC5 overexpression and recombinant irisin administration reduced apoptosis, renal dysfunction, tissue damage, tubular cell apoptosis, and inflammation during I/R. Irisin was found to inhibit p53 activation, a protein that promotes tubular cell apoptosis. These findings suggest irisin may act as a protective mechanism in renal I/R injury, indicating therapeutic potential. Similarly, Zhang et al. [175] confirmed the nephroprotective effects of irisin in animal models of acute kidney injury via I/R. According to Bi et al. [87], irisin’s role in I/R injuries involves mitochondrial protection and the mitigation of oxidative stress.

Cui et al. [176] suggest that irisin preserves mitochondrial integrity and function in renal tubular epithelial cells, and protects against I/R-induced acute kidney injury. In their study, serum irisin levels negatively correlated with creatinine levels. Post-I/R irisin administration in mice reduced markers of renal inflammation, including serum creatinine, kidney injury molecule-1 (Kim-1), and neutrophil gelatinase-associated lipocalin (NGAL). Irisin also enhanced the expression of PTEN-induced kinase 1 (PINK1), which protects cells from mitochondrial dysfunction induced by stress, supporting its protective role in renal I/R injury.

Irisin has also been shown to exert antifibrotic effects in the kidney by inhibiting the TGF-β receptor signaling pathway. In a comprehensive study, Peng et al. [177] elucidated the protective mechanism of irisin in the kidney. They used a mouse model with the muscle-specific overexpression of peroxisome proliferator-activated receptor gamma coactivator 1-alpha (mPGC1α), which acts as a physical activity substitute. These mice exhibited reduced fibrosis markers and improved renal function under stress. Serum from mPGC1α-overexpressing mice induced metabolic reprogramming in cultured renal tubular cells, increasing their oxygen consumption. mPGC1α prevented tubular damage, TGF-β1 activation, metabolic reprogramming, and raised ATP levels in injured kidneys. Subsequent analyses confirmed that irisin expression was elevated in both skeletal muscle and serum, with irisin identified as the key myokine driving adaptive changes and opposing TGF-β1-induced metabolic reprogramming, thereby benefiting renal tubules. Peng et al. [177] proposed that irisin binds to the TGF-β type II receptor (transforming growth factor beta receptor type 2, TGFBR2), thereby disrupting the TGF-β1-mediated phosphorylation of Smad2/3 proteins.

In another study, Zhou et al. [178] showed that aerobic exercise increased irisin expression in the kidneys of mice following myocardial infarction. In vitro, irisin inhibited oxidative stress and apoptosis in normal kidney cells, partially through the FNDC5/Irisin-AMPK-Sirt1-PGC-1α (irisin-amp kinase-sirtulin 1-PGC1α) signaling pathway. Liu et al. [179] also demonstrated that exercise enhances SIRT1 expression, mitigating inflammation and metabolic dysfunction in the kidneys and liver of diabetic mice.

Research suggests that physical exercise has beneficial effects for patients with kidney diseases. The nephroprotective role of physical activity may be partially attributed to irisin, which, by influencing energy metabolism, prevents or reduces kidney damage in diabetic nephropathy, acting through the irisin/AMPK axis [173]. In these studies, irisin itself activated AMPK. Han et al. [180] found that irisin reduced metabolic disturbances and protected against obesity-related CKD (OB-CKD) by acting on perirenal adipose tissue. It also modulated the VEGF-NO axis in renal glomeruli, considered a key mechanism in obesity-induced CKD.

Available evidence indicates that physical exercise positively influences kidney function through complex biological mechanisms, encompassing both improved systemic fitness and the activation of molecular pathways mediated by myokines. In particular, irisin, a myokine induced by skeletal muscle contractions during exercise, may play a key role in regulating inflammatory, metabolic, and structural processes in the kidney. The observed interactions among the above-mentioned organs further underscore the importance of the crosstalk between organs in systemic health. Although the current research findings are promising, further studies are necessary in order to fully understand the underlying mechanisms and explore their therapeutic potential.

## 8. Irisin–Lungs–Physical Exercise

Currently, relatively little is known about the function of irisin in healthy lungs [181]. Although irisin exhibits promising protective effects in pulmonary diseases, existing studies in this area are limited and require further investigation. In recent years, however, there has been a slight increase in interest regarding irisin in the context of lung tissue, as more research confirms its elevated blood concentration in response to physical exertion.

Emerging evidence suggests that irisin may play a significant role in various lung diseases [181]. Its involvement has been confirmed in the pathomechanisms of COPD, asthma, acute lung injury, pulmonary hypertension, and lung cancer. It may also serve as a biomarker and therapeutic target in pulmonary diseases. In these conditions, irisin protects cells from oxidative stress. Studies by Bi et al. [182] have demonstrated that irisin regulates and improves the function of the vascular endothelium, potentially inhibiting apoptosis and the production of inflammatory factors, and may also broaden the understanding and effectiveness of therapeutic strategies in the treatment of pulmonary diseases. Research by Shao et al. [183] suggests that irisin inhibits the proliferation and migration of cancer cells, potentially serving as a therapeutic factor in lung cancer therapy.

Irisin has also been found to play a vital role in supporting the process of autophagy. It enhances the production of the LC3-II protein, which is involved in autophagy, in response to the presence of harmful particulate matter. Elevated concentrations of airborne particulate matter are associated with increased mortality due to cardiovascular and respiratory diseases, exacerbated asthma symptoms, and impaired pulmonary function.

Aydin et al. [184] confirmed the presence of irisin in rat lung parenchyma. Immunohistochemical analyses revealed that, in alveoli, irisin expression was observed exclusively in type II pneumocytes and was absent in type I pneumocytes. Beyond the alveoli, irisin expression was also demonstrated in epithelial and smooth muscle cells of the bronchi and bronchioles, as well as in the endothelium and vascular smooth muscle cells [185].

In the latest study by Bernardes-Ribeiro et al. [186], the effects of the intracerebroventricular and intraperitoneal administration of irisin in adult male rats on cardiorespiratory and metabolic functions during sleep and wakefulness were analyzed under normoxia, hypercapnia, and hypoxia. The central administration of irisin reduced minute ventilation (VE) during wakefulness in normoxia, whereas peripheral administration decreased VE during sleep. Furthermore, the central administration of irisin intensified hypercapnia-induced hyperventilation by increasing VE and reducing oxygen consumption.

Other studies have noted that lactate produced by skeletal muscles during physical exertion, including respiratory muscles, may stimulate the secretion of irisin and brain-derived neurotrophic factor (BDNF) in the brain in a PGC-1α-dependent manner [187]. This has led to speculation that irisin and BDNF may play an important role in facilitating hyperpnea, or deepened breathing, although further research is needed [181].

Chen et al. [188] demonstrated that the remote ischemic preconditioning of limbs (RIPC) increases irisin levels in the blood and that, in conditions of ischemia-reperfusion stress, irisin penetrates damaged alveolar cells. There, it interacts with the mitochondrial protein UCP2, protecting the mitochondria and preventing lung injury. The intravenous administration of irisin alleviated lung damage in mice, although this effect was diminished in UCP2-deficient mice. Bi et al. [87] confirmed that irisin supports mitochondrial function, reduces oxidative stress, and protects the endothelial barrier, thereby mitigating inflammation and pulmonary edema. This mechanism is most likely mediated via the AMPK/SIRT1 signaling pathway [87,188,189].

Irisin and physical exercise have been confirmed to exert a mitigating effect on emphysema induced by cigarette smoking, an effect mediated via the Nrf2/heme oxygenase 1 (HO-1) pathway [190]. Shao et al. [191] indicated that irisin secreted during exercise may inhibit apoptosis and inflammation in lung epithelial cells by modulating cellular signaling pathways, including MAPK and nuclear factor kappa B (NF-κB), through which ROS are activated in lung tissue. In patients with COPD, a reduction in this factor was observed in epithelial cells of the bronchi and alveoli.

According to Sugiyama et al. [192], irisin may reduce oxidative stress caused by environmental pollution and the exposure to toxic compounds, including tobacco smoke. Similarly, the antioxidant properties of the nuclear factor erythroid 2-related factor 2 (Nrf2) have been recognized [193]. The authors suggest that the Nrf2 pathway plays a protective role in emphysema by regulating antioxidant defense, reducing pulmonary inflammation, and suppressing alveolar cell apoptosis. Irisin significantly increases Nrf2 expression and reduces risks associated with cigarette smoke exposure [192]. The same authors also confirmed that irisin may act as a mediator that limits inflammatory processes related to emphysema and plays a role in preventing disease progression. They propose that individuals with low irisin levels may be more susceptible to developing emphysema.

In a clinical study, Alyami et al. [194] conducted an eight-week supervised exercise training (SET) program in patients with interstitial lung disease (ILD). In addition to assessing respiratory parameters and physical performance, irisin concentrations were measured before and after the intervention. The study found no significant changes in irisin levels following the training, in either the patient or control groups. However, irisin levels in the patient group were higher than in the control group, which suggests that elevated irisin levels in ILD patients may result from irisin secretion by damaged lung cells as a protective response for pulmonary tissue.

Epidemiological studies indicate that COPD is one of the most prevalent respiratory diseases and a significant public health problem worldwide [195]. It considerably limits patients’ physical, emotional, and social functioning [196]. COPD is characterized by persistent airflow limitation in the respiratory tract, resulting from an inflammatory response to various toxic substances, mainly found in tobacco smoke or environmental pollutants such as dust, fumes, nitrogen oxides, and sulfur compounds that irritate the bronchial mucosa. Such irritation leads to increased mucus secretion, the hypertrophy of mucus-producing glands, and an increased number of inflammatory cells in the mucosa, which secrete substances that damage lung tissue, resulting in a narrowing of the bronchi and bronchioles and the destruction of lung parenchyma (emphysema) [194].

Inflammation exacerbates apoptosis in epithelial and endothelial cells, contributing to the development of emphysema and the progression of COPD. Low physical activity increases the risk of damage and is associated with higher mortality in COPD patients, particularly those with a low muscle mass. Therefore, regular physical activity is recommended in order to improve cardiorespiratory fitness, slow the progression of sarcopenia, and boost irisin production, which exerts beneficial effects on lung function [197].

Numerous studies confirm that cardiorespiratory fitness is significantly higher in physically active individuals than in sedentary individuals, and engaging in physical activity at every stage of life yields substantial health benefits [198,199]. It improves overall functioning, increases muscle mass, enhances cardiorespiratory efficiency, positively affects cognitive processes and mood, an important yet often underestimated aspect of this disease, and increases irisin concentration [200,201].

COPD patients are characterized by reduced physical activity, a lower quality of life, and an increased incidence of depression [202]. According to research, irisin may be a potential mediator between COPD and depression, as its expression in skeletal muscles and the brain positively correlates with physical activity. Irisin also enhances the synthesis of BDNF, which is involved in reward-related processes in the brain.

Papp et al. [196] analyzed mood disorders in COPD patients in relation to changes in irisin and BDNF synthesis, and assessed quality of life using the St. George’s Respiratory Questionnaire (SGRQ). The study found that mood disturbances correlated with serum irisin levels, which were more pronounced in patients with low BDNF levels. The authors suggest that irisin has a beneficial effect on mood in patients with COPD, likely by inducing BDNF expression in the brain as a result of physical activity.

Although irisin shows promise in the context of COPD, there remains a lack of clinical studies confirming its protective role. The disease pathogenesis involves oxidative stress, cellular aging, and inflammatory mechanisms. Due to its antioxidant properties, irisin has been recognized as a promising biomarker for COPD [203,204], while physical activity is considered a key therapeutic strategy for the disease.

Ijiri et al. [205] analyzed irisin levels in 72 COPD patients, correlating the results with pulmonary function parameters, exercise capacity, physical activity levels, and the effects of acute and chronic exercise. Compared to the control group, patients exhibited lower irisin concentrations; however, irisin levels did not correlate with any pulmonary function parameters or six-minute walk distance. However, serum irisin levels were associated with physical activity levels across all subjects. In COPD patients, acute physical exertion did not affect serum irisin levels; however, an eight-week training program resulted in a significant increase.

In a study by Sugiyama et al. [192], 40 COPD patients underwent pulmonary function testing. The authors observed lower irisin levels in patients and reported that decreased serum irisin concentrations were associated with pulmonary emphysema.

In another study, Cuttitta et al. [206] investigated the roles of body composition and the concentrations of leptin, adiponectin, and irisin in exercise capacity, respiratory function, and quality of life in COPD patients. They found that the patients’ results in the six-minute walk test (6MWT) and physical performance were significantly lower compared to the control group. A significant positive correlation was found between the leptin and fat mass, as well as between the 6MWT results and indicators of good nutritional status. A significant inverse correlation was noted between the 6MWT results and leptin and fat mass, between FEV1 and haptoglobin, and between irisin and haptoglobin. The authors confirmed the role of the body mass, body composition, and adipocytokine levels in exercise capacity, pulmonary function, and quality of life in COPD patients.

Similarly, lower irisin concentrations were reported in the blood of COPD patients compared to healthy controls in another study [207] in which 68 patients underwent pulmonary rehabilitation. Serum irisin concentrations were lower in COPD patients than in healthy individuals in the control group. After completing rehabilitation, irisin levels increased in COPD patients compared to their pre-rehabilitation levels. Examples of clinical studies proposing irisin as a biomarker in COPD are shown in Table 4.

Aside from COPD, asthma is also a common pulmonary disease. Due to its anti-inflammatory properties, irisin may also be recognized as a factor that helps limit the disease by stimulating protective mechanisms. Experimental studies indicate that, in asthma, irisin may inhibit monocyte infiltration and reduce the levels of pro-inflammatory factors such as TNF-α and IL-6 [181]. Furthermore, irisin, by regulating the function, activity, and polarization of M1/M2 macrophages through its suppressive effect on ROS production, may exert anti-inflammatory effects [208]. Unfortunately, there is still a lack of clinical studies examining the changes in irisin concentration in patients with asthma. Existing studies linking irisin and COPD are shown in Table 4. 

In summary, irisin may play a protective role in the function and pathology of the respiratory system, although its role in healthy lungs is not yet fully understood. Studies suggest that irisin contributes to the alleviation of oxidative stress, inflammation, and cellular damage in various pulmonary diseases. The observed effects indicate the potential significance of irisin as a supportive element in the treatment of lung diseases; however, further research is necessary in order to confirm its clinical applicability.

## 9. Summary and Conclusions

Based on the available data, it can be inferred that irisin, initially described primarily in the context of muscle metabolism and adipose tissue, plays a much broader, systemic physiological role that includes other organs such as the liver, kidneys, and respiratory system. Irisin exhibits protective effects in liver diseases (e.g., NAFLD), in kidney diseases (e.g., CKD), in dialysis patients, and in pulmonary conditions such as COPD, asthma, and pulmonary hypertension, through its anti-inflammatory, antioxidant, and anti-apoptotic actions. Its role as a mediator of the beneficial effects of physical exercise appears particularly significant, potentially explaining the positive impact of exercise on the course of many chronic diseases. While the preclinical study results are promising, the lack of definitive clinical evidence limits the applicability of irisin as a biomarker or therapeutic agent. Consequently, irisin represents a promising yet insufficiently understood component of the signaling pathways that maintain homeostasis, whose clinical potential requires further investigation and confirmation. Its protective role in various diseases, including those discussed—NAFLD, CKD, COPD, and asthma—as well as in sarcopenia and bone metabolism, makes irisin a promising biomarker. However, the lack of standardized and unified analytical procedures, especially those using the ELISA method, limits its practical application. The current state of knowledge on irisin necessitates that clinical test manufacturers standardize detection methodologies to ensure clarity regarding the results, as well as test sensitivity and specificity.

## Figures and Tables

**Table 1:** Selected studies on changes in blood irisin concentration after various forms of physical effort/training.

References	Group Characteristic	Type of Exercise/Training Characteristic	Irisin	Biological Material/Analytical Methods
Boström et al. [11]	Non-diabetic Individuals (n = 8 ♂)	Endurance training (cycloergometer) **S:** 20–30 min/day 4–5x/week/10 weeks **I:** ~65% VO_2_max	**↑** ** Irisin 2x **	**Plasma samples** Western blot antibodies against Fndc5 were from Abcam, Cambridge, United Kingdom
Jędrychowski et al. [17]	**Exercised** (n = 6 ♂) Age: 25 ± 5 yrs BMI = 24.3 ± 2.5 kg/m^2^ **Sedentary** (n = 4 ♂) Age: 26 ± 3 yrs BMI = 26.1 ± 3.4 kg/m^2^	HIAT ** S: ** (4 × 4 min/3x/week/12 weeks; **I:** >90% VO_2_peak 3 min rest) separated by 2x/week treadmill; **S**: 45 min; **I:** 70% peak aerobic capacity	**↑ Irisin** Sedentary: ~3.6 ng/mL Exercised: ~4.3%	**Plasma samples** Mass spectrometry with control peptides enriched with stable isotopes as internal standards
Algul et al. [59]	**Trained** (n = 60 ♂) Age: 19.2 ± 0.7 yrs BMI: 21.3 ± 0.4 kg/m^2^ **Untrained **(*n* = 30) Age: 19.5 ± 0.6 yrs BMI: 21.7 ± 0.4 kg/m^2^	All the individuals randomly performed two 30-min bouts aerobic/running I: 64–76% of their predicted maximal.	**↑ Irisin** In both groups after the morning exercise and night-time exercise	** Serum samples ** ELISA kit; Phoenix Pharmaceuticals Inc., Burlingame, CA, USA)
Hew-Butler et al. [60]	**Runners **(n = 16) 8 ♂ and 8 ♀; (>50 km/week) **Nonrunners **(n = 17) 8 ♂ and 9 ♀; (<60 min endurance activity)	10-week supervised run/walk 5 km training program	**= Irisin** (statistically no significant differences)	**Plasma samples** ELISA kit; Phoenix Pharmaceuticals, Burlingame, CA, USA
Nygaard et al. [61]	Healthy individuals Moderately trained n = 9 (7♂ 2 ♀) Age: 32 ± 9 yrs BMI: 4.5 ± 2.4 kg/m^2^ FFM: 61 ± 10 kg FAT: 24 ± 6% VO_2_max = 50 ± 7 mL/kg/min	60 min endurance (END) 60 min strength (STR) Without exercise (CON) **END:** One session of HIIT **S:** 6 × 5 min/2 min of rest; **I:** ≥18 on the Borg 6–20 scale; **STR:** Many exercises performed as three sets of 10–12RM	**↑ Irisin** Effect of intervention and time; **CON:** no change in irisin levels from pre to the other points; **END:** irisin increased at 0 h, 1 h then decreased until 24 h. **STR:** irisin increased at 1 h and tended to at 2 h 4 h then decreased, until 24 h. STR	** Plasma samples ** Measurements- 15 min prior to exercise, 0 h post-exercise, and 1 h, 2 h, 4 h, 6 h and 24 h post-exercise (END, STR); ELISA kits (Phoenix Pharmaceuticals, Inc., Burlingame, CA, USA)
Fernandez-del-Valle et al. [62]	Healthy young adults (n = 26) 14 ♂ and 12 ♀ Age: ♂ 21.18 ± 1.93/ ♀ 21.35 ± 2.52 yrs	HIRT **S:** 55 min 9x/3 weeks **I:** 70–80% 1RM	**= Irisin** compared to baseline	**Serum samples** ELISA kit; Adipogen Life Sciences, Liestal, Switzerland
Murawska—Ciałowicz et al. [63]	**Trained** (*n* = 15) **Sedentary** (Healthy subjects) (n = 10) Age: 32.39 ± 6.63 yrs	HIIT following Tabata protocol 60 min/2x/week/8 weeks; Before training period and after Wingate and GXT were made and level of irisin measured	**↑ Irisin **(29.7%) *p* < 0.05	**Serum samples** ELISA kit by BioVendor Laboratorni medicina (Brno, Czech Republic)
Shabani et al. [64]	Non-obese women **Control (n = 6)** Age: 25.50 ± 4.80 yrs **RET (n = 10)** Age 24.60 ± 2.45 yrs **AET (n = 9)** Age: 24.66 ± 2.29 yrs **CET (n = 10)** Age: 26.60 ± 4.00 yrs	**RET:** 65 min 3x/week/8 week **I:** 55–75% 1RM **AET**: step training and running; I: 60–75% age-predicted HRmax; **CET:** 25 min resistance training + 25 min aerobic training	**↓ Irisin**	**Serum samples** ELISA kit (ZellBio GmbH, Ulm, Germany)

BMI—Body mass index; S—session; I—intensity; GXT—Graded Exercise Test, HIAT—High-Intensity Aerobic Training; HIRT—High-Intensity Resistance Training; RET—Resistance exercise training; AET—Aerobic exercise training; CET—Concurrent (aerobic + resistance) exercise training; HRmax—Maximal Heart Rate; yrs—years; ↓—lower/decrease; ↑—higher/increase; ♂—male; ♀—female.

**Table 2:** Examples of clinical studies that used irisin as a non-alcoholic fatty liver disease biomarker.

Study	Disease	Group	Irisin/ Analytical Methods/ Biological Material	Effects/ Comments
Kosmalski et al. [121]	NAFLD+ T2DM	138 patients—70♂/68 ♀ Age: 65.61 ± 10.44 yrs; (n = 72) In NAFLD group (n = 72), 46 patients with T2DM In without NAFLD group (n = 66), 31 patients with T2DM	**↑ Irisin** in NAFLD patients/ ELISA Kit (BioVendor—Laboratorní medicína a.s. Brno, Czech Republic)/ Serum samples	Irisin corelated with BMI, HbA1c, AST, Cr, urea NAFLD risk with irisin level above 3.235 μg/mL was 4.57 times higher than in patients with lower level of irisin
Nayak et al. [122]	T2DM with NAFLD	T2DM patients (n = 90) Healthy controls (n = 90) Age: 30–55 yrs Patients divided into 4 groups: T2DM with NAFL T2DM without NAFLD Controls with NAFLD Controls without NAFLD	**↓ Irisin** in patients with T2DM compared to health controls. Patients with T2DM with NAFLD had ↓ irisin levels than those without NAFLD/ ELISA kit (Biocodon Technologies, Mission, KS, USA)/ Serum samples	Significant correlations of irisin levels with insulin sensitivity markers such as HOMA-IR and QUICKI across different groups
Ulualan et al. [123]	NAFLD	60 pubertal obese children (31 ♀, 29 ♂), Age: 11–18 yrs 30 of them had NAFLD Control group—healthy children (n = 28; 28 (14 ♀, 14 ♂)) similar in age and sex to the obese group	**↓ Irisin** (median) in the obese than in control group; **↓ Irisin** (median) in patients with and without NAFLD in comparison to control. No differences between irisin level in patients with and without NAFLD/ ELISA kits (BioVendor Inc., Candler, NC, USA)/ Serum samples	Irisin negatively correlated with BMI, waist, hip and arm circumferences, waist/hip ratio, skinfold thickness, and AST and ALT levels
Shanaki et al. [124]	NAFLD T2DM	NAFLD (n = 41) T2DM (n = 41) NAFLD + T2DM (n = 40) Control (n = 40)	**↓ Irisin** in NAFLD, T2DM and NAFLD+T2DM compared to control; **↑ Irisin** correlated with reduce risk of T2DM, NAFLD and NAFLD+T2DM; **↓** levels of irisin cannot be associated with T2DM and NAFLD; ELISA kit (BioVendor, Brno, Czech Republic)/Plasma samples	Irisin levels negatively correlated with BMI, WHR, visceral fat, HOMA-IR, FBG, insulin, liver stiffness, and liver enzymes; based on multiple stepwise linear regression, ALT and irisin level were independent predictors for liver stiffness
Polyzos et al. [125]	NAFL NASH	NAFL (n = 15) NASH (n = 16) Control lean (n = 24) Control obese without NAFLD (n = 28)	**↓ Irisin** in obese controls and patients with NAFL and NASH compared with lean controls; there were no differences between irisin level in patients with NAFL, NASH, and obese controls/ELISA kit (Phoenix Pharmaceuticals, CA, USA)/Serum samples	Difference remained significant after adjustment for BMI (or waist circumference), gender, age, insulin resistance (assessed by HOMA-IR or QUICKI), exercise, and time since blood collection
Li et al. [126]	T2DM concomitant with MAFLD	T2DM without MAFLD (n = 80) MAFLD without T2DM (n = 62) T2DM with MAFLD (n = 50) Healthy (n = 80)	**↓ Irisin** in patients with T2DM with MAFLD compared to T2DM and MAFLD alone ELISA kit R&D Systems, USA) Serum samples	Irisin’s level ↓ as the levels of FPG and FINS increased
So et al. [127]	NAFLD	NAFLD (n = 274) Healthy volunteers (n = 37) Subjects with NAFLD divided into 4 groups according to physical activity levels and body adiposity (by BMI = 30): Inactive and Non-obese (n = 99) Inactive and Obese (n = 51) Active and Non-obese (n = 85) Active and Obese (n = 39) 124 active subjects took part in intervention study with a 12-weeks weight loss program	**↓ Irisin **in NAFLD than in healthy volunteers **↑ Irisin **in active groups than in inactive ELISA kit Serum samples	Hepatic steatosis level inversely correlated with irisin levels In the weight loss program, subjects with ↑ irisin levels had a great reduction in fatty mass, subcutaneous adipose area, γGTP, leptin, and TNFα

BMI—Body Mass Index; HbA1—Glycosylated Hemoglobin; AST—Aspartate Transaminase; ALT—Alanine Transaminase; Cr—Creatinine; ↓—lower/decrease; ↑—higher/increase; WHR—Waist-to-Hip Ratio; HOMA-IR—Homeostatic Model Assessment of Insulin Resistance; QUICKI—Quantitative Insulin Sensitivity Check Index; FBG—Fasting Blood Glucose; FINS—Fasting Insulin; γ-GTP—γ-Glutamyl Transpeptidase; ♂—male; ♀—female.

**Table 3:** Examples of clinical studies that used irisin as a biomarker for chronic kidney disease.

Study	Disease	Group	Irisin/ Analytical Methods/ Biological Material	Effects/ Comments
Liu et al. [167]	T2DM with and without kidney insufficiency	Patients with T2DM across a wide range of renal function (n = 365)	**↓ Irisin **in T2DM with renal insufficiency (77.4 ± 13.7 ng/mL in T2DM with eGFR ≥ 60 mL/min/1.73 m(2) versus 72.5 ± 14.9 ng/mL in those with eGFR < 60 mL/min/1.73 m(2), *p* = 0.001) ELISA kit Phoenix Pharmaceuticals Inc (Burlingame, CA)	Reduction in irisin level was most pronounced in stage 5 CKD patients. Irisin in T2DM with preserved renal function correlated with age, and pulse pressure. In patients with renal insufficiency, irisin was correlated with BMI, fat mass, percentage of fat mass, and eGFR.
Rodríguez-Carmona et al. [168]	CKD	CKD with peritoneal dialysis or with hemodialysis (n = 95) Healthy control (n = 40)	**↓ Irisin **in all CKD groups in comparison to control. Furthermore, patients with peritoneal dialysis had higher serum level of irisin than those on hemodialysis and those managed conservatively ELISA kits Adipogen International, San Diego, USA)	Limited correlations between irisin, on the one hand, and fat (but not lean) mass, GFR, and plasma albumin and bicarbonate. Plasma bicarbonate and GFR identified as independent predictors of irisin levels.
Sadeghi Shad et al. [169]	CKD	CKD patients in stage 2 and stage 4 (n = 90) Stage 2 (n = 45) Stage 4 (n = 45)	**↓ Irisin** in patients in stage 4 compared with patients at stage 2 (ELISA) kit (Zell BioGmbH, Germany	Serum irisin, GFR, Alb, HDL and Hb levels significantly ↓ Inversely, Cr, TG, LDL, FBS, BUN, and urea significantly ↑ from stage 2 to stage 4. These findings suggest that irisin may be involved in the regulation of biochemical factor levels in CKD.
Shelbaya et al. [170]	T2DM	T2DM (n = 60) Healthy subjects (n = 30) T2DM patients divided: Without diabetic complications (n = 30) With diabetic nephropathy (DN) (n = 30)	**↓ Irisin** in diabetic patients compared to controls **↓ Irisin** in diabetic patients with DN compared to those without complications	There was a statistically significant negative correlation between irisin and serum Cr, SBP, DBP duration of diabetes, BMI albumin/creatinine ratio, and HbA1c in all T2DM patients. Duration of diabetes was the only independent determinant of irisin level.

T2DM—Diabetes Mellitus type 2; CKD—Chronic Kidney Disease; eGFR—glomerular filtration rate estimated; BMI—Body Mass Index; Alb—Albumin, HDL—High-Density Lipoproteins; Cr—Creatinine; TG—Triglyceride; LDL—Low-Density Lipoproteins; FBS—Fasting Blood Sugar (Glucose), SBP—Systolic Blood Pressure; DBP—Diastolic Blood Pressure; HbA1c—Glycosylated Hemoglobin; ↓—lower/decrease; ↑—higher/increase.

**Table 4:** Examples of clinical studies using irisin as a COPD biomarker.

Study	Group	Irisin/Analytical Methods/ Biological Material	Effects/Comments
Papp et al. [196]	COPD (n = 74) Quality of life was evaluated by Saint George’s Respiratory Questionnaire (SGRQ)	**↓ Irisin** in COPD patients with mood disturbances (ELISA) kit (Phoenix Pharmaceuticals, Burlingame, CA, USA)/Serum samples	Association between low irisin level and mood disturbances was stronger among patients with the lower level of BDNF and weaker in the higher level of BDNF.
Ijiri et al. [205]	COPD patients (n = 72) Healthy control (n = 27) 8 weeks of physical training	**↓ Irisin** in COPD patients than in healthy subjects (ELISA) kit (Phoenix Pharmaceuticals, Burlingame, CA, USA)/Serum	Irisin not correlated with pulmonary function parameters. Irisin level associated with physical activity level in all subjects. 8-week training was linked to increase in irisin level.
Sugiyama et al. [192]	COPD patients (n = 40)	**↓ Irisin** in COPD patients ELISA kit Phoenix Pharmaceuticals, Burlingame, CA, USA/Serum	Decreased serum irisin levels are related to emphysema in patients with COPD and involved.
Cuttitta et al. [206]	COPD patients (n = 25) Smokers and nonsmokers Healthy subjects (n = 26)	**↓ Irisin** in COPD patients who are smokers than in nonsmokers ELISA kit (Cusabio, Houston, TX, 77054, USA)	Irisin/muscle mass ratio higher in non-smokers vs. smokers and in females vs. males.
Ma et al. [207]	Patients with COPD Before and after pulmonary rehabilitation (n = 68) Healthy control (n = 35)	**↓ Irisin** in COPD patients than in healthy control group ELISA/serum	↑ Irisin after physiotherapy rehabilitation.

COPD—Chronic Obstructive Pulmonary Disease; BDNF—Brain-Derived Neurotrophic Factor; ↓—lower/decrease; ↑—higher/increase.

## Data Availability

No new data were created or analyzed in this study.
